# Harmonizing hybridization dissonance in conservation

**DOI:** 10.1038/s42003-020-1116-9

**Published:** 2020-07-21

**Authors:** Claudio S. Quilodrán, Juan I. Montoya-Burgos, Mathias Currat

**Affiliations:** 1 Department of Zoology, University of Oxford, Oxford, United Kingdom; 2 Laboratory of Anthropology, Genetics and Peopling History, Anthropology Unit, Department of Genetics and Evolution, University of Geneva, Geneva, Switzerland; 3 Laboratory of Vertebrate Evolution, Department of Genetics and Evolution, University of Geneva, Geneva, Switzerland; 4 Institute of Genetics and Genomics in Geneva (IGE3), Geneva, Switzerland

**Keywords:** Biodiversity, Conservation biology

## Abstract

A dramatic increase in the hybridization between historically allopatric species has been induced by human activities. However, the notion of hybridization seems to lack consistency in two respects. On the one hand, it is inconsistent with the biological species concept, which does not allow for interbreeding between species, and on the other hand, it is considered either as an evolutionary process leading to the emergence of new biodiversity or as a cause of biodiversity loss, with conservation implications. In the first case, we argue that conservation biology should avoid the discussion around the species concept and delimit priorities of conservation units based on the impact on biodiversity if taxa are lost. In the second case, we show that this is not a paradox but an intrinsic property of hybridization, which should be considered in conservation programmes. We propose a novel view of conservation guidelines, in which human-induced hybridization may also be a tool to enhance the likelihood of adaptation to changing environmental conditions or to increase the genetic diversity of taxa affected by inbreeding depression. The conservation guidelines presented here represent a guide for the development of programmes aimed at protecting biodiversity as a dynamic evolutionary system.

## Abstract (web summary)

In this Perspective article, Claudio Quilodrán et al. discuss how hybridization should be considered with respect to conservation. They argue hybridization may be useful for species conservation by enhancing the ability of taxa to adapt better to changing environmental conditions or increasing genetic diversity.

## Introduction

Biodiversity is in crisis and the main reasons are human activities inducing habitat modifications and the introduction of invasive species^1^. In addition, global climate change will probably alter habitat characteristics, migration patterns, reproduction time, and place of various species^2^. Such human disturbances may produce new breeding overlaps, breaking the independent evolution of organisms and leading to hybridization (see Glossary, Table 1)^3^. The role of hybridization in the evolution of several plant and animal taxa has been recognized in the light of newly developed molecular tools^4^. This has also alerted biologists about the threat this phenomenon may represent to biodiversity when enhanced by anthropogenic factors^5^. We identified three types of hybridization regarding the reproductive properties of first-generation hybrids (F_1_). This is proposed as a framework to investigate the demographic and genetic effects of hybridization on biodiversity.

**Table 1:** Glossary.

Concept	Definition
Adaptive introgression	Maintenance of introgressed alleles due to natural selection
Admixture	Genomic mixing resulting from the interbreeding between genetically distinct groups
Demographic flow	Clonal production of parental types through hybrid offspring in hybridization with genome exclusion
Gene flow	Transfer of genes between different groups of organisms
Genome exclusion	Fertile hybrid offspring that exclude one parental genome during gametogenesis
Genomic mixing	New genetic architecture in hybrid populations due to the recombination of homologous chromosomes during gametogenesis
Hybridization	Interbreeding between genetically distinguishable groups, e.g., from different populations, species or genera
Hybridogenesis	Hybridization with genome exclusion
Inbreeding depression	Fitness reduction in a population induced by the mating between closely related individuals
Interbreeding	Mating between any genetically distinct group of organisms
Introgression	Gene flow from one genetically distinct group to another, due to hybridization
Outbreeding depression	Fitness reduction of hybrids relative to parental individuals
Speciation reversal	Loss of genetic distinctiveness due to hybridization with introgression

Our perspectives come from the development of modeling simulation approaches applied to various real case studies, which helped us to explore the outcomes of hybridization from both conservation and evolutionary perspectives. We bring here a novel view of conservation guidelines aiming to state the conditions under which hybridization may represent priorities for conservation programmes or, alternatively, new evolutionary opportunities. We highlight that hybridization may certainly lead to biodiversity loss when enhanced by human factors, leading for instance to outbreeding depression or the introgression of maladaptive genes. However, it may also drive the emergence of new biodiversity, reducing the effects of inbreeding depression, and increasing the opportunities to adapt to changing environmental conditions.

### Species concept problem and interspecific hybridization

The widely accepted biological species concept formulated by Mayr^6^ states that species are “*groups of actually or potentially interbreeding natural populations which are reproductively isolated from other such groups*”. The key idea under this vision is the reproductive isolation that delimits the species unit. This was already proposed by Georges-Louis Leclerc, Comte de Buffon, more than 260 years ago^7^. Buffon realized that a horse and a donkey are morphologically more similar than some different races of dogs. However, the reproduction in the first case leads to an infertile offspring (a mule) while in the second case, the offspring is fertile, highlighting that a line can be drawn between organisms that cannot reproduce in order to differentiate species.

Charles Darwin supported a different view and dedicated an entire chapter of “*On the Origin of Species*” to the hybridization concept^8^. The observation of interbreeding between distinct morphological species, with different degrees of offspring fertility, from completely sterile to even more fertile than parental species in determined conditions, was an argument against sterility or fecundity as a species delineation factor. Darwin agreed with the notion that species may hardly remain different when free sympatric mating occurs, but supported a more continuous conception of species, influenced by the gradual effect of natural selection. However, the idea of species with various degrees of fertility was abandoned during the modern evolutionary synthesis^6,9,10^.

Much of the understanding about reproductive isolation and interspecific hybridization has been revealed by experimental studies of *Drosophila*^11^. Those works revealed that: (i) reproductive isolation is positively correlated with the phylogenetic distance between hybridizing species; (ii) at the same level of genetic divergence, reproductive isolation is higher between sympatric than allopatric species; and (iii) hybrid offspring follow Haldane’s rule, meaning that if one sex is less viable or sterile, it is more likely to be the heterozygotic sex^12,13^. During most of the 20th century, interspecific hybridization was considered to be rare in nature, mainly arising by human translocation of species and with a small effect in evolution, with hybrids supposedly having lower fertility in most cases^14^.

Despite the wide acceptance of reproductive isolation as a key element to define species, a large controversy persists around the biological species concept (Box 1). This is mainly motivated by the semipermeable breeding barriers between some species and the difficulty of testing this notion in organisms with nonoverlapping spatial or temporal distribution ranges^15,16^.

Box 1 Alternative species concepts Three of the most popular alternative definitions of species are the ecological, phylogenetic, and evolutionary concepts. Ecological species are of closely related lineage using minimally differentiated adaptive zones, also denominated as ecological niches^93^. Evolutionary species are defined as ancestral-descendant lineages with their own identity, evolutionary tendency and historical fate^94^. Phylogenetic species are in turn considered to be the minimal cluster of organisms with a pattern of ancestry and descendance^95^. These three definitions have also been criticized. The ecological and evolutionary species concepts have been judged to be too vague to determine a cut-off point between species^15,17^. The phylogenetic species concept has been defended by various authors in the field of conservation biology, who consider it an encompassing view of unique ancestral and derived features for separate species, e.g., refs. ^15,19^. However, this definition has also been the focus of criticism, mainly due to an inflated number of species^16^. This is because different regions of the genome may express very different evolutionary histories and because hybridization may also perturb phylogenetic classifications by altering monophyletic lineages^20^. Mallet^96^ recognized various cases of speciation that are influenced by fertile hybridization in nature and tried to rescue and adapt the more continuous view of species proposed by Charles Darwin. He understood species as groups of genotypes that remain distinct in the face of actual or potential hybridization^96,97^. He highlighted the fact that genotypes may remain distinct with reproductive isolation, but this would be a way to maintain species or to reach speciation rather than being a means of species discrimination^96^. To date, there are around 30 definitions of species and a large debate about species concept and the relation with hybridization, e.g., refs. ^15,17,18,98^.

### Species concept and conservation

A problematic view arises when applying the biological species concept, which does not make room for interspecific hybridization^17^. The semipermeable barriers between genetically, morphologically or ecologically distinct organisms have motivated a large debate about species and hybridization, e.g., refs. ^15,18^. This discussion is not superfluous for conservation biology because it delimits the main unit of protection^17^. Yet, what are the central criteria to delineate the units that deserve protection? Some authors consider that because species are evolutionary units, the most appropriate way to diagnose them objectively is through the phylogenetic species concept^19^. But the use of the phylogenetic species concept has been criticized because small, isolated populations may become well diagnosed evolutionary lineages through the effect of strong genetic drift, inflating the number of species and rendering protection actions more complex. Other authors have advocated that the criteria to delineate conservation units should rely on evidence of reproductive isolation or reduced reproductive fitness^20^, but these criteria are less objective and sometimes difficult to evidence.

The debate about species concept and hybridization is not only a matter for biologists, but also for scientists from very different domains, as well as politicians who define legal aspects of wildlife protection^21^. In this sense, Pasachnik et al.^22^ propose that whatever else a species is, in the field of conservation biology it should be a group of organisms deserving legal protection because its extinction would constitute a meaningful loss of biodiversity. The evolution of biodiversity represents a continuum, in which speciation processes may occur slowly or relatively fast, but will always have a period of uncertainty regarding genetic differentiation between emerging species^23^. Conservation biology may therefore consider the level of uncertainty due to hybridization by protecting biodiversity as a dynamic system, which is not focused on reproductive isolation to delimit discrete units, but on the sum of features for which the loss of certain organisms may represent a detrimental effect on biodiversity.

### Evolution of new biodiversity

Botanists first highlighted the important role of natural hybridization on the speciation process of several species, i.e., in generating new biodiversity, e.g., ^24,25^. Later, zoologists recognized the major evolutionary effects of introgression on numerous insects e.g., ref. ^26^, fishes, e.g. ref. ^27^, amphibians, e.g., ref. ^28^, reptiles, e.g., ref. ^29^, birds, e.g., ref. ^30^, mammals, e.g., ref. ^31^; and other organisms, e.g., ref. ^32^, including modern humans (Box 2). There are around 25% of plants and 10% of animals that are currently known to hybridize with another species and the effect of this phenomenon in evolution is considered to be much more important than previously thought^33^.

Species can naturally change their historical home range in response to changing environmental conditions and meet closely related taxa^34^. Several species carry signatures of hybrid ancestry from the last Ice Age period, e.g., ref. ^27^. For this reason we can find DNA of brown bears in polar bears, because ancient hybridization events occurred during the Pleistocene^35^. The Bering Land Bridge recurrently emerged during this time, allowing organisms to migrate between Eurasia and North America, leading to opportunities of hybridization, such as those observed between Canada lynx (*Lynx canadensis*) and Eurasian lynx (*Lynx lynx*)^31^. Organisms can have introgressed genes from locally extinct species even if they have never been in contact, because a third species, acting as a temporal bridge to gene flow, has hybridized with both of them e.g., ref. ^36^.

Natural selection may fix beneficial alleles obtained by hybridization or, to the contrary, remove detrimental introgressed alleles. Adaptive introgression has been important for several speciation processes^33^. For instance, the antipredatory mimicry of three *Heliconius* butterflies in South America has been acquired by interspecific hybridization, for which the parts of the genome related to color patterns have more introgressed alleles than other regions of the genome^37^. Introgressed alleles can rapidly spread among individuals when they are related to adaptive traits. For example, “warfarin” is a rodenticide that was developed in 1948 to control house mice (*Mus musculus*). Mice started to be resistant during the 1960s by acquiring a single gene from the Algerian mouse (*M. spretus*) through hybridization^38^. These species were isolated until the development of human agricultural lands. They rarely interbreed and hybrids have limited survival with half of them being sterile, but the resistance gene rapidly spread across Europe. In Germany, where both species do not mingle, one third of house mice have the introgressed resistance gene coming from Algerian mice^38^. A similar case was documented between two species of mosquitos that are vectors of malaria and have different levels of resistance to an insecticide^39^. The insecticide acted as a selective pressure driving the spread of resistant alleles obtained by hybridization, even when hybrids had reduced fertility^40^. The reduced fertility of the offspring is therefore not necessarily selected against and can also represent adaptive mate choice^41^.

Opportunities for speciation as a result of hybridization can be generated when hybrids exploit unique ecological niches. For instance, a rapid incipient speciation was recently observed in the offspring of two species of yeast, *Saccharomyces paradoxus* and *S. cerevisiae*, whose hybrids have the potential to exploit a unique ecology that is intermediate between those of the parental species^32^. The new genetic architecture generated by hybridization can thus also facilitate ecological divergence, promoting a speciation process by exploiting a specific niche, e.g., ref. ^42^.

**Fig. 1:**
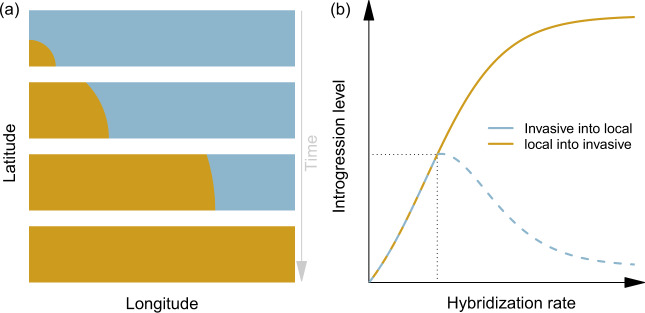
Expected pattern of introgression of neutral genes between local and invasive organisms in range expansion. **a** The context of this expected pattern of introgression is the expansion of an invasive species (in beige) in an area where the local species (in blue) is already in demographic equilibrium. The invasive species starts its colonization from the bottom left side of the area with few individuals. **b** The level of introgression is asymmetrical and higher in the invasive organisms when the interbreeding rate is large enough (after the dotted line in the *x*-axis). The value of the admixture rate that delineates this expected higher introgression in the invasive taxon depends on the combination of demographic and migration parameters^43^. The introgression asymmetry between the two species is due to local alleles continuously introduced at the wave front of the invasive range expansion, with a relatively high probability of increasing in frequency due to the surfing process^44^. The invasive organisms are not necessarily non-indigenous and may also represent threatened organisms that increase in frequency at the expense of exotic organisms^45^.

Positive selection can fix adaptive alleles and purifying selection can remove the detrimental alleles, e.g., ref. ^27^, but introgressed genes can remain even without the effect of natural selection. Neutral introgressed alleles can persist in high proportion, even when the original species is extinct. Currat et al.^43^ demonstrated through computer simulations and by a review of the literature, that invasive species in range expansion may carry a large quantity of neutral alleles that are introgressed from a local species. The reverse is not necessarily true unless interbreeding is rare (Fig. 1). When hybridization occurs during the expansion of an invasive species into the territory of a local species, introgression is indeed expected to be much higher in the invasive species than in the local species (Fig. 1). This pattern of asymmetric introgression is generally robust to the density and population structure within both species and to the level of interspecific competition. It results from the hybridization level and from the population demographic imbalance at the wave front of the invasion, in which introgressed alleles that are continuously introduced in the invasive species along its expansion, may surf and reach a higher frequency than expected under a stationary context^44^. While this pattern may be perturbed by density-dependent dispersal^45^ and long-distance dispersal^46^, there are several real cases of asymmetrical introgression between demographically imbalanced species that have been proposed to follow this neutral expectation, e.g. refs. ^47,48^.

Box 2 Hybridization and human evolution Hybridization has probably also played a role in our own evolution when modern humans spread out of Africa and met other closely related hominids. Analyses of ancient DNA estimated around two percent of Neanderthal ancestry in the genome of modern humans outside Africa^99–101^. The introgressed genes may have persisted through neutral processes^102^ or as a result of positive selection e.g., ref. ^103^. Recently, it has been proposed that some introgressed alleles, adaptive in the past, may currently be associated with certain diseases^104^. Modern humans are likely to have met and potentially interbred with other hominids in addition to Neanderthals. Huerta-Sánchez et al^105^. recognized positive selection in haplotypes related to survival at high altitudes in current Tibetans, which seem to have been introgressed from Denisovans. Other haplotypes from Denisovan ancestry seem to be frequent in the current genome of Melanesians^106^. Our own genome may thus carry the result of various ancient hybridization events during human evolution^107^.

### Biodiversity loss

Hybridization is considered as a major conservation concern when it is motivated by anthropogenic factors, such as translocation of invasive species or by modification of natural habitats^5,49^. The breakdown of the reproductive barriers between organisms may disrupt their independent evolution and has already increased the risk of extinction of several plant and animal taxa, e.g., refs. ^50,51^.

Hybridization may lead to different but potentially interacting mechanisms that threaten species persistence. First, outbreeding depression may represent a significant loss of reproductive value and detonates a rapid extinction when it interacts with a demographic decline. This may be stronger between genetically distant species e.g., ref. ^52^, but organisms do not need to be distantly related to be affected by outbreeding depression. For instance, the human domestication of Atlantic salmon (*Salmo salar*) has led to lower fertility when mating with conspecifics in the wild, representing a serious threat for wild salmon in Norway^53^. Second, native genotypes can disappear by genetic swamping and be replaced by the numerical or competitive advantage of invasive genotypes. Third, the introgression of non-native genes can disrupt local adaptations by introducing maladaptive gene complexes^54^. Fourth, the behavior of wild animals may be perturbed in a way that is difficult to predict, more particularly when it concerns human domesticated animals^55^, which have been artificially selected according to human lifestyle and, when spreading their genes in nature, may influence a whole network of ecological interactions, e.g., ref. ^56^. Fifth, hybridization may affect the effective population size of the interacting species with major consequences for rare or threatened species, which already have a reduced number of breeders^57^. Finally, an important problem for conservation biology arises when the few remaining individuals of a threatened species show a level of introgression that may cause them to lose their legal protection status when hybrids are not considered to be protected organisms, even though the hybrids may have an ecological function otherwise lost with the extinction of parental species^21,58^.

The loss of species distinctiveness due to introgression has also been called “speciation reversal”, e.g., ref. ^59^. This may seriously affect key ecological adaptations that appeared during species radiation. Vonlanthen et al.^60^ documented the rapid extinction of whitefish (*Coregonus* spp.) in Swiss lakes, which evolved according to ecological opportunities, but human eutrophication and homogenization of the environment is driving extinction by hybridization and demographic decline. A similar case was documented for cichlid fishes of Lake Victoria (East Africa), for which the coloration pattern is a key character that determines mate choice and reproductive isolation, but the turbidity of the water induced by eutrophication relaxed sexual selection, destroying the diversification mechanism^61^. Speciation reversal is a conservation concern, because it erodes the ecological and genetic distinctiveness between closely related, but ecologically divergent, species^60^. In a context of climate change, Owens and Samuk^62^ refers to hybridization as a double edge sword, because even when increasing the pool of potentially adaptive genes, some of these genes may be related to reproductive isolation, weakening any reproductive barrier. The various cases of hybridization leading speciation reversal, e.g., refs. ^59,61^, suggest that the extinction risk may be more extensive than previously thought^60^.

Hybridization between wild and domesticated organisms is a worldwide problem of conservation. For instance, the main current threat for the persistence of European wildcats (*Felis silvestris*) is the hybridization with domestic cats (*Felis catus*)^63,64^. Domestic cats were originally domesticated from a wildcat inhabiting the Near East (*Felis lybica*), but they are genetically distinct to all current *F. lybica* subspecies^65^. There are still some wildcat populations remaining in Europe, e.g., ref. ^66^, but the complete admixture and the loss of genetic distinctiveness have already been achieved in some countries^67^. Domestic dogs (*Canis familiaris*) can hybridize with any kind of wolf-like canids and have already led to conservation issues in various cases^50^, such as for the gray wolf (*Canis lupus*) in Europe, e.g., ref. ^68^ the coyote (*Canis latrans*) in North America, e.g., ref. ^56^ or the Ethiopian wolf (*Canis simensis*) in Africa, e.g. ref. ^69^. Ellington and Murray^56^ found that hybridization with domestic dogs was driving changes in the space occupied by coyotes, suggesting consequences at the ecosystem level. A particular threat is the hybridization of domestic dogs with the Ethiopian wolf, which is the world’s most endangered canid, persisting with around 500 individuals in 6 isolated populations^69,70^. The detrimental effects of hybridization with domesticated organisms is reinforced, because they far outnumber their wild counterparts, e.g., ref. ^71^, in which the extinction risk can be particularly accelerated when rare species hybridize with more abundant species.

Genetically modified organisms and genetic engineering have generated a large debate on how to regulate the spread of modified genes in nature through hybridization e.g., ref. ^72^. Genomic alteration for economic purposes may induce higher fertility and resistance to pathogens that make crops or hybrids highly invasive^73^. The reduced fertility of the first-generation hybrids (F_1_) is not a barrier for the spread of advantageous alleles^74^, which are frequently observed in the wild, e.g., ref. ^75^ with hybrids becoming invasive in various cases^76^. The ecological release of their natural predators or pathogens conferred by the resistant alleles has been proposed as a factor that is initiating this invasion^73^. A serious risk has been detected in the single wild population of rice in Costa Rica (*Oryza glumaepatula*) that hybridizes with invasive commercial rice (*O. sativa*)^77^. The concerns are not only related to modified plant crops, but also to animals of economic interest, usually with unpredictable ecological effects, e.g., ref. ^78^ or to non-target insects, as has been documented for the monarch butterfly *Danaus plexippus* of North America, e.g., ref. ^79^.

### Types of hybridization

**Fig. 2:**
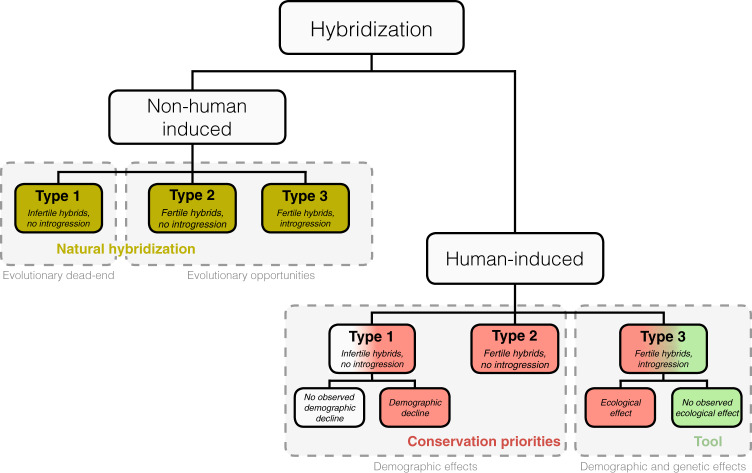
Three types of hybridization regarding the reproductive characteristics of first-generation hybrids (F_1_). Type 1 represents infertile or inviable hybrids. Type 2 hybrids are fertile but introgression is prevented in further generations due to the generation of gametes without recombination during gametogenesis in hybrid offspring. Type 3 hybrids are fertile and there is recombination during gametogenesis allowing introgression in further generations. Non-human-induced hybridization represents hybrids naturally found in nature, in which evolutionary opportunities arise when hybrids are fertile. Conservation guidelines are proposed for human-induced hybridization, which are motivated by any anthropogenic factor. They represent either a purely demographic or both a demographic and genetic effect on interbreeding taxa. The conservation priorities to avoid biodiversity loss are highlighted in red and basically represent human-induced hybridization that produces demographic decline or ecological disequilibrium. A potential tool to increase genetic diversity is highlighted in green.

We defined three main types of hybridization that may be used as a framework for the understanding of the ecological and evolutionary consequences of hybridization (Fig. 2). These categories include: (1) distant species hybridization, mostly preventing gene flow because hybrids are infertile (Type 1) or (2) because homologous chromosomes do not recombine (Type 2); and (3) interbreeding between more closely related taxa, in which homologous chromosomes recognize themselves during meiosis, resulting in gene flow and consequent introgression between parental organisms (Type 3) (Box 3).

**Fig. 3:**
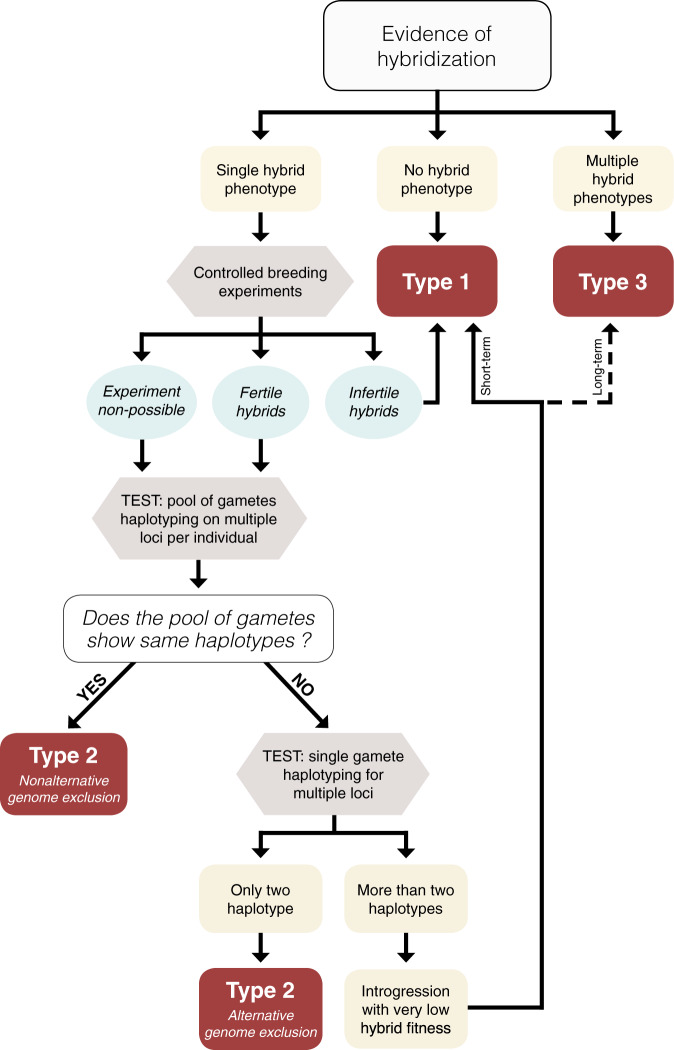
Identification of the type of hybridization. Different steps that may be considered to recognize the type of hybridization when there is evidence of interbreeding between taxa (modified from Quilodrán et al.^81^).

#### Type 1: Infertile hybrids, no introgression

The first type of hybridization does not result in introgression, because offspring are inviable or infertile. This type of hybridization represents an extinction risk when the loss of reproductive value enhances a demographic decline for one (or both) parental species. The reasons could be either because small populations interbreed with more abundant populations and therefore waste reproductive efforts, or because additional threats are accumulated, such as a disease. For example, in the case of hybridization between Atlantic salmon (*Salmo salar*) and brown trout (*Salmo trutta*), hybridization alone is likely not a threat, but could lead to the extinction of some local salmon populations that are already threatened by a parasitic disease^80^. This type of hybridization may be considered an evolutionary dead-end.

#### Type 2: Fertile hybrids, no introgression

The second type of hybridization results in fertile F_1_ hybrids, but introgression is prevented because their offspring are clonal or hemiclonal, transmitting a single parental genome, also called hybridization with genome exclusion. We recently showed that the extinction of natives and the invasion of exotic organisms might be reached in very few generations^81^. For instance, in the case of hybridogenesis between Western European water-frogs (*Pelophylax* species complex)^51^, the extinction risk is not genetically driven, but determined by the “demographic flow” between parental species and mediated by hybrid offspring. We previously demonstrated that this hybridization is a highway to extinction, which may be underappreciated because it emulates the result of hybridization type 1 (i.e., only displaying F_1_ hybrid phenotypes)^81^. Evolutionary opportunities may emerge from these systems by generating self-reproducing polyploid forms^82^, which are observed in plants but rarely found in animals^83^.

#### Type 3: Fertile hybrids, introgression

The third type of hybridization defines interbreeding with gene flow between parental organisms leading to genomic mixing and therefore to introgression. This type of hybridization may result in two different effects on biodiversity, either a genetic and demographic risk of species extinction^5^, or the opportunity of adaptation and evolution of novel diversity^14^. For instance, hybrids may replace native species and facilitate biological invasions as in the case of mallard (*Anas platyrhynchos*), which has been widely translocated, cohabiting with other duck species and threatening them by hybridization^84^. In another example, however, genes from extinct hominids may still be found in high frequency in current human populations due to old hybridization events^43,85^. This type of hybridization can also represent a new evolutionary opportunity by increasing genetic diversity and possibilities of adaptation^84^.

Box 3 Assignment to hybridization type The three types of hybridization constitute a useful guideline for the understanding of the genetics and/or demographics effects of hybridization on biodiversity. However, to determine one of these types in a specific real system is not always an easy task to achieve, especially when it regards the past evolution of already extinct organisms or when it regards the projection of long-term effects. For instance, infertile hybrids, but with very small introgressions, are observed between Atlantic salmon and brown trout^108^. A small level of introgression may be ignored, when there is a short-term effect of hybridization producing extinction risk^80^, but it would not be the case when projecting evolutionary long-term effects, and even more so when concerning range expansions (see Fig. 1), in which case it would be considered as type 3. Moreover, detection of hybridization type 3 with low levels of introgression strongly depends on the amount of genetic markers evaluated^4^.

Because hybridization type 1 and type 2 are both producing only F_1_ phenotypes, we recently developed a genetic framework to determine the type of hybridization^81^ (Fig. 3). Type 3 is easier to recognize due to multiple hybrid phenotypes being present in a population caused by different levels of introgression. If only F_1_ phenotypes are observed, often with a phenotype intermediate between parental taxa, we recommend defining hybrid fertility by performing controlled breeding experiments. If these experiments are not possible because, for instance, the few remaining individuals of the involved species are threatened, it would be useful to observe their demography and sex ratio. Hybridization type 2 generally produces a very fast demographic decline, and most of the time favors the production of a single sex (namely females). When hybridization type 2 is suspected, it is important to define whether hybrids’ gametes are producing a single (non-alternative) or both (alternative) parental genomes. This may be clarified with a pool of gametes haplotyping test, which will show whether all gametes of an individual have a single allele per gene, in which case hybridization type 2 will be of the non-alternative form. If two alleles are present for some loci, this test reveals hybridization type 2 of the alternative form. In this last case, a single gamete haplotyping method may be implemented to determine the proportion of gametes generated from each of the parental taxa. If those tests result in more than two haplotypes, it would indicate introgression with very low fitness: either type 1 when regarding ecological short-term effects or type 3 when considering evolutionary long-term effects. Details about the pool of gametes and single gametes haplotyping test are presented in Quilodrán et al.^81^.

### Conservation guidelines

Allendorf et al.^49^ proposed hybridization categories that are widely used to prioritize conservation actions. They considered three categories, but defined differently than ours: (i) sterile hybrids, (ii) widespread introgression, and (iii) complete admixture. Indeed, they ignore the effect of fertile hybrids without introgression (hybridization type 2), which is the category that may induce faster extinctions. In addition, they considered the anthropogenic motivation *a sine qua non* condition to distinguish the conservation issues of hybridization. We highlight here that hybridization, even when induced by humans, is potentially representing a source of genetic variation that could be useful for conservation purposes.

The classification of Allendorf et al.^49^ has been employed during the past 20 years, but the wider understanding of hybridization impact brought by more recent studies allows us to propose a novel view of conservation priorities (Fig. 2). Given our classification, the conservation priorities are also found in human-induced hybridization, but this is not the single cut-off to delimit them. Hybridization type 1 is a conservation concern when promoting demographic decline, either because two species with high density-imbalance interbreed or because hybridization amplifies other existing risks^80^. Hybridization type 2 is always a threat that may precipitate extinction within very few generations^81^. Hybridization type 3 is also a priority when affecting key ecological interactions, either by enhancing demographic decline or because it changes the behavior of wild individuals^84^.

Hybridization types 1 and 3 should not represent a priority when they are not triggering demographic decline or the disruption of ecological functions^80,84^. We suggest that the resources to protect biodiversity may be redirected either to other conservation issues or other threatened organisms. In such conditions, hybridization type 3 may even be used as a conservation tool to increase genetic diversity. However, all of these should be implemented carefully^84^. The potential fitness loss and the detrimental ecological effects of hybridization have first to be evaluated, and this is often difficult to achieve. In the first case, controlled breeding experiments may help to assess the fertility of hybrids. If this is not possible, monitoring the demography of parental species may help to evaluate a potential fitness loss due to hybridization. A detrimental ecological effect of hybridization is more difficult to evaluate but the behavior of hybrids may provide valuable information. As an example, in Britain, extent hybridization has been registered between Scottish wildcats and domestic cats^86^, as well as between European polecat and feral ferrets^87^. While the phenotype of Scottish wildcats has been seriously affected^86^, the polecat phenotype has been much less affected due to hybridization^87^. In both cases, the increased genetic diversity may have a positive effect in front of changing environmental conditions, but the impact of hybridization on the behavior of wildcats^55^, and on the fitness of the polecat population^88^, deserve more attention before rejecting hybridization as a threat or proposing it as a conservation management tool.

We propose that phylogenetically closer taxa with similar ecological requirements may offer some guidelines for assisted hybridization as a tool in conservation. For instance, assisted hybrization between subspecies of panthers has promoted the recovery of Florida panthers (*Puma concolor coryi*) by increasing heterozygosity and decreasing inbreeding, resulting in an overall increase of survival and fitness^89^. Hybridization between different species has also promoted the recovery of American chestnuts (*Castanea dentata*) through the transfer of pathogen resistance from Chinese chestnuts (*C. mollisima*)^90^. In circumstances where organisms are evolutionarily close and share similar ecologies, and when the local species is on the brink of extinction, hybrids may also represent a subject of protection, even when hybridization is caused by anthropogenic factors. An example is the interspecific hybridization between coral reefs, in which the parental species *Acropora palmata* and *A. cervicornis* have been in a critical decline over the last decades, but their hybrids (also called *A. prolifera*) have increased in several locations^91^. The hybrids have been shown to be as fit or even more fit than the parental species^92^. While the parental species are legally protected, protecting hybrids represents a legal challenge, which may help to preserve functional ecosystems otherwise lost with the extinction of the parental species^91^.

## Conclusions

Hybridization that influences both the loss and the creation of new biodiversity may seem paradoxical at a first glance. The loss of native biodiversity is certainly an issue related to conservation biology when it is induced by anthropogenic factors under the conditions exposed in Fig. 2. However, hybridization had influenced the evolution of several species of hybrid origins, e.g., refs. ^26,31^, participating in the creation of novel biodiversity. This is therefore not a real paradox, but an intrinsic property of hybridization, which may drive the extinction of native species and at the same time stimulate the appearance of new species. The conservation guidelines defined here constitute an important framework to understand the ecological and evolutionary consequences of hybridization. The conservation priorities established in Fig. 2 are not delimitated only by human hyphen induced origins of hybridization, but by the disruption of key ecological interactions driven by genetic and demographic factors. This highlight that hybridization, even when induced by humans, may also represent a subject of protection. This classification notably incorporates the effect of hybridization type 2, which was previously ignored^49^. We propose that it should be considered as a potential highway to extinction, and thus deserves high priority in conservation programmes.
